# Prevalence, evaluation and management of overactive bladder in primary care

**DOI:** 10.1186/1471-2296-10-8

**Published:** 2009-01-23

**Authors:** Wellman W Cheung, Nadia H Khan, Karmina K Choi, Martin H Bluth, Miriam T Vincent

**Affiliations:** 1 Department of Urology, SUNY Downstate Medical Center, Brooklyn, New York, NY, USA; 2 Department of Pathology, Wayne State University/Detroit Medical Center, Detroit, MI, USA; 3 Department of Family Practice, SUNY Downstate Medical Center, Brooklyn, New York, NY, USA

## Abstract

**Background:**

Patients with overactive bladder (OAB) are under-diagnosed in the primary care setting. Primary care physicians (PCP) approach to the patient and appropriate patient disclosure may contribute to under-diagnosis.

**Methods:**

An outpatient primary care setting was used to determine the prevalence and characteristics of OAB. Patients who visited the family medicine outpatient clinic were invited to answer a self-administered questionnaire. It included questions on evidence of lower urinary tract symptoms (modified Overactive Bladder-Validated 8-question Screener [OAB-V8]), relevant medical and surgical history, and demographic data. Relationship between OAB and other independent variables were analyzed using chi-square and risk ratio (RR) analysis.

**Results:**

Of 325 questionnaires distributed, 311 were returned completed. Patients ranged from 18 to 97 years, the majority women (74.0%) and African American (74.3%). OAB was present in 60.5% of men and 48.3% of women (*p *= 0.058). OAB was significantly associated with obesity (BMI > = 30) in women (*p *= 0.018, RR = 1.72), specifically obese premenopausal women (age < 55 years) (*p *= 0.011, RR = 1.98).

**Conclusion:**

OAB prevalence is more than double and higher in men than previously reported. The relative risk for OAB is significantly greater in obese premenopausal women.

## Background

Overactive bladder (OAB) is a common disorder that negatively affects the quality of life [1,2] of our patients and carries a large socioeconomic burden [3]. OAB is a condition caused by sudden involuntary contraction (over-activity) of the bladder detrusor muscles. According to the International Continence Society (ICS), it is characterized as urinary urgency, with or without urge incontinence, usually with frequency and nocturia, in the absence of causative infection or pathological conditions [4]. On the basis of a population-based survey of 16 776 men and women aged 40 years and older conducted by Milsom *et al *[5] in 6 countries, by telephone or direct interview, the prevalence of OAB in Europe has been estimated to be 15.6 and 17.4% for men and women respectively, with an overall prevalence of 16.6%. In the NOBLE survey in which 11740 Americans agreed to participate, overall prevalence of OAB was 16.9% in women and 16.0% in men [2].

It is expected that the primary care practitioner (PCP) will identify and initiate the management of patients with OAB [6]. Despite its high prevalence, many afflicted patients remain undiagnosed or untreated [5,7]. The prevalence of OAB and its evaluation and management in primary care has never been studied in the US. Moreover, little epidemiologic data exists for OAB in ethnic/minority groups.

## Methods

In a two week period, 325 patients aged 16 years and above who visited one of two family medicine outpatient centers, for non-urological issues, were invited to answer a self-administered questionnaire on urinary symptoms while waiting to be seen by their PCPs; physicians also completed a survey regarding their experience of patients with urinary symptoms in their practice (Additional files 1 and 2). 311 patients agreed to fill the questionnaire. This study was approved by the Institutional Review Board, SUNY Downstate Medical Center, Brooklyn, NY.

The modified patient questionnaire included 8 questions on evidence of lower urinary tract symptoms (LUTS) (Overactive Bladder-Validated 8-question Screener [OAB-V8]) [8], 2 questions on stress urinary incontinence (SUI), and one question on incomplete emptying of the bladder during urination. Responses were answered on a 6-point Symptom Bother scale ranging from 0 (not at all) to 5 (a very great deal). An additional question asked about the effect of urinary symptoms on quality of life (QOL), with answers ranging from 0 (delighted) to 6 (terrible). The questionnaire also asked for data on demographic characteristics and relevant medical and surgical history. Patient weight and height were measured for calculating body mass index (BMI) (kg/m^2^).

Patients were considered to have OAB if their OAB-V8 score is ≥ 8 [8]. The presence of specific LUTS was then analyzed. Patients with OAB were categorized into subtypes based on their presence of particular symptoms. The categorization scheme and criteria for LUTS and OAB subtypes are shown in Table 1. Patients who did not complete the OAB-V8 and SUI questions were excluded from the study. For the purpose of our analysis, patients were categorized into age groups of < 25, 25–34, 35–44, 45–54, 55–64, 65–74, and ≥ 75. Women were considered to be pre-menopausal if age < 55 years, and postmenopausal if age ≥ 55.

**Table 1:** Criteria for LUTS and OAB subtypes

	*Idenitifcation criteria*
*LUTS*	
Urgency	Answer ≥1 to any one of the three OAB-V8 questions on urge to urinate
Frequency	Answer ≥1 to any one of the two OAB-V8 questions on frequent urination
Nocturia	Answer ≥1 to the OAB-V8 question on nocturia
Urge urinary incontinence (UUI)	Answer ≥1 to one of the two OAB-V8 questions on urine leakage associated with urge
Stress urinary incontinence (SUI)	Answer ≥1 to one of the two questions on urine leakage associated with physical activities, sneezing, coughing, or laughing
Mixed urinary incontinence (MUI)	Meet the criteria for both UUI and SUI described above
Incomplete emptying	Answer ≥1 to the question on incomplete emptying
*OAB subtypes (based on presence of above symptoms)*	
OAB with frequency alone (OAB-F)	OAB-V8 score ≥8 with symptoms of frequency and/or nocturia only
OAB with urgency without incontinence (OAB-U)	OAB-V8 score ≥8 with symptoms of urgency without incontinence
OAB with urge urinary incontinence (OAB-UUI)	OAB-V8 score ≥8 with symptoms of UUI and no SUI
OAB with stress urinary incontinence (OAB-SUI)	OAB-V8 score ≥8 with symptoms of SUI and no UUI
OAB with mixed urinary incontinence (OAB-MUI)	OAB-V8 score ≥8 with symptoms of MUI

LUTS = Lower urinary tract symptoms

Unadjusted age- and sex-specific prevalence of OAB and sex-specific prevalence of LUTS and OAB subtypes were reported. The chi-square test was used to determine statistically significant differences between the two sexes. To determine the relationship between OAB prevalence and other factors (race, menopausal status, BMI, history of smoking and history of hysterectomy), covariates were first individually evaluated using the chi-square test. Statistically significant (*p *< .05) covariates were retained for risk ratio analysis. Patients were excluded from a specific analysis if they did not report on the variable to be analyzed. Results are presented as risk ratio and 95% confidence interval (RR, 95% CI).

All statistical analyses were performed using SPSS 15.0 for Windows.

## Results

Table 2 reports the characteristics of patients studied including age, sex, ethnicity, education, BMI, smoking, prior surgical history and OAB status. Patients ranged from 18 to 97 years, the majority were women (74.0%) and Black (74.3%). Of the 219 women who reported their age, 49.3% were postmenopausal. OAB was present in 60.5% men and 48.3% women (*p *> .05). Prevalence of OAB increased with age in men but did not vary with age in women.

**Table 2:** Characteristics of participants

Characteristic, *n *(%)	Male (*n *= 81)		Female (*n *= 230)		Total (*N *= 311)	
Age, years						
< 25	2	(2.5)	14	(6.1)	16	(5.1)
25–34	5	(6.2)	27	(11.7)	32	(10.3)
35–44	9	(11.1)	28	(12.2)	37	(11.9)
45–54	20	(24.7)	42	(18.3)	62	(19.9)
55–64	16	(19.8)	41	(17.8)	57	(18.3)
65–74	20	(24.7)	42	(18.3)	62	(19.9)
≥75	8	(9.9)	25	(10.9)	33	(10.6)
Unknown	1	(1.2)	11	(4.8)	12	(3.9)
Race						
Black	57	(70.4)	174	(75.7)	231	(74.3)
Hispanic	13	(16.1)	27	(11.7)	40	(12.9)
White	0	(0.0)	3	(1.3)	3	(0.01)
Other/Not stated	11	(13.6)	26	(11.3)	37	(11.9)
Education						
Less than high school	8	(9.9)	13	(5.7)	21	(6.8)
High school graduate	24	(29.6)	72	(31.3)	96	(30.9)
Some college/college graduate	19	(23.5)	47	(20.4)	66	(21.2)
Graduate school	4	(4.9)	13	(5.7)	17	(5.5)
Not stated	26	(32.1)	85	(37.0)	111	(35.7)
Body mass index (BMI), kg/m^2^						
< 25.0	12	(14.8)	38	(16.5)	50	(16.1)
25.0–29.9	27	(33.3)	79	(34.3)	106	(34.1)
≥30	31	(38.3)	96	(41.7)	127	(40.8)
Not stated	11	(13.6)	17	(7.4)	28	(9.0)
History of smoking*						
Yes	36	(44.4)	59	(25.7)	95	(30.5)
No	45	(55.6)	171	(74.3)	216	(69.5)
Previous surgical history						
Bladder surgery**	0	(0.0)	8	(3.5)	8	(2.6)
Urinary leakage surgery**	0	(0.0)	1	(0.4)	1	(0.3)
Hysterectomy***	--	--	49	(21.3)	--	--
Prostate surgery	5	(6.2)	--	--	--	--
OAB status						
Normal	32	(39.5)	119	(51.7)	151	(48.5)
OAB with UI	37	(45.7)	92	(40.0)	129	(41.5)
OAB without UI	12	(14.8)	19	(8.3)	31	(10.0)

Analysis of the 151 patients without OAB (119 women, 32 men) shows that women reported 53.8% frequency and 44.5% nocturia. Urgency was present in 25.2% of the 119 women, with UUI in 23.3% of these women. SUI was present in 25.2% of the 119 women. Among the men without OAB there were less reported lower urinary tract symptoms (31.3% frequency, 46.9% nocturia and 12.5% urgency).

Table 3 identifies the prevalence of LUTS and OAB subtypes in men and women with OAB. Each of the three OAB-defining symptoms, urgency, frequency, and nocturia, were present in almost 90% of OAB patients. UUI was highly prevalent in both genders, however more so in women (79.3% vs 75.5%, *p *> .05). SUI was significantly more prevalent in women (60.4% vs 36.7%, *p *= .006). Incomplete emptying, a symptom often accompanying OAB, was present in about half OAB patients and was similar in both men and women (53.1% vs 47.7%).

**Table 3:** Prevalence of LUTS and OAB subtypes by gender

*n *(%)	Male (*n *= 49)	Female (*n *= 111)	*p *value
Symptoms			
Urgency	44 (89.8)	100 (90.1)	.954
Frequency	48 (98.0)	110 (99.1)	.550
Nocturia	41 (83.7)	101 (91.0)	.177
UUI	37 (75.5)	88 (79.3)	.595
SUI	18 (36.7)	67 (60.4)	.006
Incomplete emptying	26 (53.1)	53 (47.7)	.535
OAB subtypes			
OAB-F	1 (2.0)	4 (3.6)	.600
OAB-U	11 (22.4)	15 (13.5)	.158
OAB-UUI	20 (40.8)	25 (22.5)	.018
OAB-SUI	0 (0.0)	4 (3.6)	.178
OAB-MUI	17 (34.7)	63 (56.8)	.010
OAB with incontinence	37 (75.5)	92 (82.9)	.277

UUI = urge urinary incontinence, SUI = stress urinary incontinence, OAB-F = OAB with frequency alone,

OAB-U = OAB with urgency without incontinence, OAB-UUI = OAB with urge urinary incontinence,

OAB-SUI = OAB with stress urinary incontinence, OAB-MUI = OAB with mixed urinary incontinence

The sex-specific differences in OAB subtypes found in this study reveal that the OAB-MUI subtype was significantly more common in women (56.8% women vs 34.7% men, *p *= .010) and OAB-UUI was significantly more common in men (40.8% vs 22.5%, *p *= .018). OAB-U was also more prevalent in men, and men were less likely to have OAB with incontinence than women (*p *> .05).

**Figure 1:**
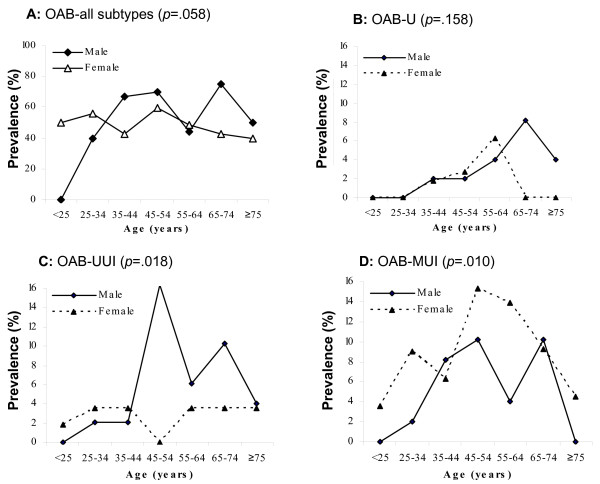
**Prevalence of OAB subtypes by age and sex**. Data represent percent (%) prevalence of male and female patients by age for (A) all subtypes, (B) OAB-U, (C) OAB-UUI, (D) OAB – MUI. OAB-U = OAB with urgency without incontinence, OAB-UUI = OAB with urge urinary incontinence, OAB-MUI = OAB with mixed urinary incontinence.

Age- and sex-specific prevalence of OAB and OAB subtypes are shown in Figure 1. Prevalence of OAB increased with age in men but not in women (Figure 1A). Prevalence of OAB-U increases steadily after age 30 in both genders and peaks at age 60 for women and at age 70 for men (Figure 1B). For OAB-UUI, the prevalence in men increases from 0% at age 20 to a peak of 16.3% by age 50 (Figure 1C). OAB-UUI prevalence in women is lower than men in all age groups except those women younger than 40. For OAB-MUI, the prevalence is higher in women at most ages (Figure 1D).

Unexpectedly, OAB was present in 53.2% pre-menopausal women and 44.4% postmenopausal women (Table 4). Premenopausal women have a higher prevalence of OAB-MUI (64.4% vs 52.1%, *p *> .05) than post-menopausal women with OAB. Postmenopausal women have a higher prevalence of OAB-U (18.8% vs 10.2%, *p *> .05) and OAB-UUI (25.0% vs 15.3%, *p *> .05).

**Table 4:** Prevalence of LUTS and OAB subtypes by menopausal status

*n *(%)	Premenopause (*n *= 111)	Postmenopause (*n *= 108)	*p *value
OAB positive	59 (53.2)	48 (44.4)	.197
OAB subtypes			
OAB-F	2 (3.4)	2 (4.2)	.833
OAB-U	6 (10.2)	9 (18.8)	.204
OAB-UUI	9 (15.3)	12 (25.0)	.207
OAB-SUI	4 (6.8)	0 (0)	.066
OAB-MUI	38 (64.4)	25 (52.1)	.198
OAB with incontinence	51 (86.4)	37 (77.1)	.208

OAB-F = OAB with frequency alone, OAB-U = OAB with urgency without incontinence,

OAB-UUI = OAB with urge urinary incontinence, OAB-SUI = OAB with stress urinary incontinence,

OAB-MUI = OAB with mixed urinary incontinence

OAB was significantly associated with obesity (BMI ≥ 30) in women (*p *= .018) (Table 5). However when menopause status was controlled, such association was only found in obesity pre-menopausal women (*p *= .013), who had a 2-fold increased risk for OAB (RR = 1.98, 1.05–3.74). Overall, the risk for obese women to have OAB is 1.72 times (95% CI 1.04–2.84) that of women with normal weight. OAB is significantly associated with incomplete bladder emptying (*p *< .001) and with SUI (*p *< .001) in both men and women. OAB was not shown to vary with race, cigarette use, history of hysterectomy, menopause status in women or BMI in men (*p *> .05).

**Table 5:** Relative risk for OAB by BMI category

	Risk Ratio (RR)**		
Comparison group*	RR	95% CI	*p*
	
All women			
BMI 25.0–29.9	---	---	.091
BMI ≥30.0	1.72	1.04 – 2.84	.018
Premenopausal women			
BMI 25.0–29.9	---	---	.394
BMI ≥30.0	1.98	1.05 – 3.74	.011
Postmenopausal women			
BMI 25.0–29.9	---	---	.072
BMI ≥30.0	---	---	.188

*Women in the corresponding age group with BMI < 25 served as the reference group

** Risk ratio was calculated only when *p *< .05;

Risk ratio = prevalence of OAB in women with comparison BMI/prevalence of OAB in women with reference BMI of < 25

## Discussion

In this study we investigated the prevalence of OAB, its evaluation in primary care using a patient questionnaire. We found an OAB prevalence that is more than double that reported in a recent US population-based study [2], a finding not previously reported [2,5,7]. Variations from earlier studies may be attributed to the methodology of data collection and our non-traditional minority, predominantly female target population. This study used a clinically validated questionnaire [8] with added questions focusing on stress urinary incontinence to identify OAB patients in primary care. Unlike two population-based studies [2,7] in which responses regarding LUTS were elicited from non-institutionalized individuals through telephone interviews, our questionnaire relies on self-report by primary care physicians and outpatients. In this sense, our data collection method resembles one office-based PCP study [7], which had an OAB prevalence estimate that was high (26.5%) and most nearly resembles our results. In all three previous studies cited above, OAB was defined based on the presence and/or frequency of specific LUTS, and the study populations were predominately white. Our study differs in that the OAB criteria are based on scoring on a symptom bother scale and the patient population is predominately black. These differences may explain in part the discrepancy in OAB prevalence observed in the current study compared to those previously reported in the literature.

Significantly more men compared to women with OAB have OAB-UUI in this study. In addition, OAB with incontinence is more common than OAB without incontinence in our study in both men and women. These results are in sharp contrast to data reported previously [2], which showed women to be more likely to have OAB-UUI, and men to be more likely to have OAB without incontinence. However, when UUI is taken as a symptom, the higher prevalence we found of UUI and SUI in women than in men are consistent with other studies [2,7]. Most studies on female UI have reported SUI as more common than UUI,[7] especially in younger women,[9,10] while MUI was the major presentation overall [7,9,10]. We find that most women who have OAB have OAB-MUI. However, UUI and OAB-UUI are more prevalent than SUI and OAB-SUI in women with OAB in our study. This finding is supported by others [11,12] who reported black women to have lower rates of SUI but higher rates of UUI than white women. The significant association between OAB and SUI we found is also supported [10]. While most studies have reported white women to have higher risk for UI in general [9,13,14], how this would affect the characterization of OAB prevalence in such population could be the focus of future investigation.

As in earlier reports [2,5,7], we find OAB prevalence to increase with advancing age, however this relationship is observed only in men and we do not understand why this association is not found in women. We also find a significant association between OAB and incomplete emptying of the bladder in both men and women. Bladder outlet obstruction leading to incomplete emptying is commonly seen in benign prostate hyperplasia (BPH) in older men. Studies have reported that OAB often coexists with BPH [15], and the associated incomplete emptying of the bladder as a cause of OAB has been discussed in the clinical scenario of BPH [16]. However, the relation between OAB and incomplete emptying in women may involve mechanisms other than obstruction [16].

Obesity as a risk factor for OAB [2,10] and UI [9,14,17] has been previously reported. The risk for OAB we found for premenopausal obese women (1.98) is similar to that reported by Stewart et al [2] (2.2) in obese women with OAB-UUI. In concordance with results reported by Stewart et al, we do not find an association between OAB and obesity in men or in the general population studied. There has been no report of the relationship between smoking and OAB, and smoking is shown as a risk factor for UI in some studies [14,17] but not others [9]. We do not find any significant association for OAB and smoking in men or women.

Previous studies have demonstrated that hysterectomy increased the risk for UUI [18] but not SUI [18,19]. Hysterectomy is thought to contribute to UI through pelvic nerves or pelvic floor damage. Its relationship and contribution to OAB has not been studied, and we do not find any significant association between OAB and hysterectomy in pre- or postmenopausal women.

A number of limitations should be considered when interpreting the results of this study. Language barriers and cultural differences in how comfortable patients would voluntarily answer particular questions are challenges in implementing a questionnaire-based study. Measures have been taken to ensure that questionnaires were distributed to all qualified patients who visited the clinic, with the intention to minimize overestimation of prevalence. In our present study the response rate was 95%. The majority of our OAB patients have all 3 cardinal symptoms (frequency, nocturia, and urgency/UUI), suggesting that they have OAB and should be evaluated and managed as such.

## Conclusion

The high prevalence of OAB found in this study suggests that the needs of many of our patients with OAB may not be met by their primary care providers. Increasing the awareness and knowledge of OAB among PCPs and adopting effective clinical approaches such as a reliable, valid and "user-friendly" screening questionnaire may serve to both allow patients with urinary symptoms to comfortably report their bladder related complaints and increase the ability of PCP's to recognize, evaluate and treat OAB. Future studies should focus on differentiating OAB patients across various ethnic groups and in diverse clinic settings. Identifying culturally sensitive strategies for evaluating and managing primary care patients with OAB may help to decrease the burden of OAB and better serve our patients.

## Competing interests

The authors declare that they have no competing interests.

## Authors' contributions

Patient and data recruitment (WC, NK, KC, MV). Manuscript preparation (WC, NK, KC, MB, MV). Data and statistical analysis (WC, NK, KC, MB, MV). All authors read and approved the final manuscript.

## Pre-publication history

The pre-publication history for this paper can be accessed here:



## Supplementary Material

Additional file 1 **Physician Survey.** Click here for file

Additional file 2 **Urinary Symptom Questionnaire.** Click here for file
