# Supplementary material for: Prevalence, evaluation and management of overactive bladder in primary care
Source: BMC Fam Pract. 2009 Jan 23;10:8. doi: 10.1186/1471-2296-10-8 (PMC2642771; doi:10.1186/1471-2296-10-8)
Supplement: Additional file 1 — Physician Survey. [file 1471-2296-10-8-S1.doc]

**Physician Survey**

Name: ____________________________________ Job title: Attending / Resident Date: ____________

**Please give your best estimates to the following questions:**

1. How many patients do you see per week? _______________
2. What you do perceive to be the prevalence of urinary symptoms among your patient population?

0-10% 41-50% 81-90%

11-20% 51-60% 91-100%

21-30% 61-70%

31-40% 71-80%

1. What percentages of patients you see have urinary symptoms?

0-10% 41-50% 81-90%

11-20% 51-60% 91-100%

21-30% 61-70%

31-40% 71-80%

1. For those patients who have urinary symptoms, please give a breakdown of their age and gender:

Age range % male % female

20-29 _______ ________

30-39 _______ ________

40-49 _______ ________

50-59 _______ ________

60-69 _______ ________

70-79 _______ ________

80 and above _______ ________

*(all percentages, male plus female, should add up to 100)*

1. Who usually brings up the urinary symptoms? You (the physician) The patient Both
2. What are the most common presenting urinary symptoms? Please list all (at least three):

__________________________________________________________________________________________________________________________________________________________________________________________________________________________________________________________________

______________________________________________________________________________________

**Please answer questions 7-11 using the scale to the right:**

**Never Rarely Sometimes Often Frequently**

1. How often do you see patients that have urinary symptoms? 1 2 3 4 5
2. How often do you ask new patients about urinary symptoms? 1 2 3 4 5
3. How often do you ask established patients who do not have

preexisting urinary problems about urinary symptoms? 1 2 3 4 5

**Never Rarely Sometimes Often Frequently**

1. How often do you treat patients with urinary symptoms? 1 2 3 4 5
2. How often do you refer patients with urinary symptoms

to specialists:

a) before you initiate any treatment? 1 2 3 4 5

b) after you have treated patient and treatment failed? 1 2 3 4 5

c) overall? 1 2 3 4 5

1. What specialists do you refer your patients with urinary symptoms to?

To urologists ________% of the time

To OB/GYN ________% of the time

To other specialists ________% of the time

*(all percentages should add up to 100)*

1. If you treat patients’ urinary symptoms:

a) What are the treatments you most frequently use? (Please check all that apply)

Biofeedback

Bladder diary

Kegel exercise

Medical management—alpha adrenergic agonists (pseudoephedrine)

Medical management—alpha blockers

Medical management—anticholinergic agent

Medical management—5-alpha-reductase inhibitor

Medical management—estrogen (oral)

Medical management—estrogen (topical)

b) What is the overall success rate for treating? __________ %

c) How long do you treat before considering that treatment has failed? _____________________________

1. What is your level of confidence in evaluating and treating patients with urinary symptoms?

1 - Not confident at all

2 - Not very confident

3 - Average

4 - Somewhat confident

5 - Very confident

1. How helpful do you think a screening questionnaire would be in identifying urinary symptoms?

1 - Not helpful at all

2 - Not very helpful

3 - Average

4 - Somewhat helpful

5 - Very helpful

1. Please feel free to provide additional comments:

__________________________________________________________________________________________________________________________________________________________________________________________________________________________________________________________________
