# Supplementary material for: Prevalence, evaluation and management of overactive bladder in primary care
Source: BMC Fam Pract. 2009 Jan 23;10:8. doi: 10.1186/1471-2296-10-8 (PMC2642771; doi:10.1186/1471-2296-10-8)
Supplement: Additional file 2 — Urinary Symptom Questionnaire. [file 1471-2296-10-8-S2.doc]

# Urinary Symptom Questionnaire

*For office use*:

New patient: __Yes __No

Weight: ____________ kg

Height: ____________ cm

Did you fill out this survey before? ___Yes ___ No

Sex: M / F Your Age: ________

Ethnicity: African American / Asian / Hispanic / White / Others: ___________________________

Place of birth: ________________________ Number of years in the U.S.: _______ years

Level of education: Grade 8 or below / High school / College / Graduate school or above

Please circle the number that best describes **how bothered have you been by**…

|  | | | | | | | | | | | | | |
| --- | --- | --- | --- | --- | --- | --- | --- | --- | --- | --- | --- | --- | --- |
|  | Not at all | | A little bit | | Some-what | | Quite  a bit | | A great deal | | A very great deal | |  |
|  | | | | | | | | | | | | | |
| 1. Frequent urination during the daytime hours? | 0 | | 1 | | 2 | | 3 | | 4 | | 5 | |  |
|  | | | | | | | | | | | | | |
| 1. An uncomfortable urge to urinate? | 0 | | 1 | | 2 | | 3 | | 4 | | 5 | |  |
|  | | | | | | | | | | | | | |
| 1. A sudden urge to urinate with little or no warning? | 0 | | 1 | | 2 | | 3 | | 4 | | 5 | |  |
|  | | | | | | | | | | | | | |
| 1. Accidental loss of small amounts of urine? | 0 | | 1 | | 2 | | 3 | | 4 | | 5 | |  |
|  | | | | | | | | | | | | | |
| 1. Frequent urination in the evening? | 0 | | 1 | | 2 | | 3 | | 4 | | 5 | |  |
|  | | | | | | | | | | | | | |
| 1. Waking up from sleep because you had to urinate? | 0 | | 1 | | 2 | | 3 | | 4 | | 5 | |  |
|  | | | | | | | | | | | | | |
| 1. An uncontrollable urge to urinate? | 0 | | 1 | | 2 | | 3 | | 4 | | 5 | |  |
|  | | | | | | | | | | | | | |
| 1. Urine loss associated with a strong desire to urinate? | 0 | | 1 | | 2 | | 3 | | 4 | | 5 | |  |
|  | | | | | | | | | | | | | |
| - - - 1. Urine loss associated with physical activities, such as lifting heavy objects or exercising? | 0 | | 1 | | 2 | | 3 | | 4 | | 5 | |  |
|  | | | | | | | | | | | | | |
| 1. Urine loss associated with sneezing, coughing, or laughing? | 0 | | 1 | | 2 | | 3 | | 4 | | 5 | |  |
|  | | | | | | | | | | | | | |
| 1. Feeling you are unable to empty your bladder completely? | 0 | | 1 | | 2 | | 3 | | 4 | | 5 | |  |
|  | | | | | | | | | | | | | |
|  | | | | | | | | | | | | | |
|  |  |  | |  | |  | |  | |  | |  |  |
|  | Delighted | Pleased | | Mostly satisfied | | Mixed | | Mostly dissatisifed | | Unhappy | | Terrible |  |
|  | | | | | | | | | | | | | |
| 1. If you were to spend the rest of your life with your urinary condition just the way it is now, how would you feel about that? | 0 | 1 | | 2 | | 3 | | 4 | | 5 | | 6 |  |
|  |  |  | |  | |  | |  | |  | |  |  |

***Please continue on the other side***

How many children do you have? _______

Have you ever had surgery of the bladder? ____ No ____ Yes

Have you ever had surgery for urinary leakage? ____ No ____ Yes

Have you ever smoked? ____ No ____ Yes

Have you ever had surgery of the prostate? (Male only) ____ No ____ Yes

How many times were you pregnant? (Female only) _______

Have you ever had hysterectomy? (Female only) ____ No ____ Yes 🡪 ___ Total hysterectomy

___ Partial hysterectomy

**Thank you for filling out the questionnaire.**

***For physician use:***

Please indicate **for the current visit**:

Exit diagnosis: (list all)________________________________________________________________________

Any urinary symptom detected before reviewing the questionnaire? ____ Yes ____ No

Any urinary symptom detected after reviewing the questionnaire? ____ Yes ____ No

Will you treat patient for the urinary symptom(s)? ____ Yes ____ No

Is patient referred to a specialist? ____ Yes ____ No (urologist / GYN / others_______________)

Comments:____________________________________________________________________________________________________________________________________________________________________________________________________________________________________________________________________________________________________________________________________

Physician’s Name: __________________________ (attending / resident /nurses /PA /other________________)
